# Nutrition Transition and Biocultural Determinants of Obesity among Cameroonian Migrants in Urban Cameroon and France

**DOI:** 10.3390/ijerph14070696

**Published:** 2017-06-29

**Authors:** Emmanuel Cohen, Norbert Amougou, Amandine Ponty, Juliette Loinger-Beck, Téodyl Nkuintchua, Nicolas Monteillet, Jonathan Y. Bernard, Rihlat Saïd-Mohamed, Michelle Holdsworth, Patrick Pasquet

**Affiliations:** 1 Centre National de la Recherche Scientifique, Unité Mixte Internationale 3189, Environnement, Santé, Société, Faculté de Médecine-Nord, 51 bd Pierre Dramard, 13344 Marseille CEDEX 15, France; 2 Centre National de la Recherche Scientifique, Unité Mixte de Recherche 7206, Eco-Anthropologie et Ethnobiologie, Musée de l’Homme, Muséum National d’Histoire Naturelle, 17 place du Trocadéro, 75016 Paris, France; namougou@mnhn.fr (N.A.); amandineponty@gmail.com (A.P.); jloinger@gmail.com (J.L.-B.); nkuintchua@yahoo.fr (T.N.); nmonteillet@free.fr (N.M.); jonathan.bernard@inserm.fr (J.Y.B.); rihlat@mnhn.fr (R.-S.M.); ppasquet@mnhn.fr (P.P.); 3 MRC/Wits Developmental Pathways for Health Research Unit, Department of Paediatrics, Faculty of Health Sciences, University of the Witwatersrand, 7 York Road, Parktown, Johannesburg 2193, South Africa; 4 School of Health and Related Research, Public Health section, The University of Sheffield, Sheffield, Regent Court, 30 Regent Street, Sheffield S1 4DA, UK; michelle.holdsworth@sheffield.ac.uk

**Keywords:** nutrition transition, Cameroon, France, migrants, determinants, obesity

## Abstract

Native of rural West Cameroon, the Bamiléké population is traditionally predisposed to obesity. Bamiléké who migrated to urban areas additionally experience the nutrition transition. We investigated the biocultural determinants of obesity in Bamiléké who migrated to urban Cameroon (Yaoundé), or urban France (Paris). We conducted qualitative interviews (*n* = 36; 18 men) and a quantitative survey (*n* = 627; 266 men) of adults using two-stage sampling strategy, to determine the association of dietary intake, physical activity and body weight norms with obesity of Bamiléké populations in these three socio-ecological areas (rural Cameroon: *n* = 258; urban Cameroon: *n* = 319; urban France: *n* = 50). The Bamiléké valued overweight and traditional energy-dense diets in rural and urban Cameroon. Physical activity levels were lower, consumption of processed energy-dense food was frequent and obesity levels higher in new migrants living in urban Cameroon and France. Female sex, age, duration of residence in urban areas, lower physical activity and valorisation of overweight were independently associated with obesity status. This work argues in favour of local and global health policies that account for the origin and the migration trajectories to prevent obesity in migrants.

## 1. Introduction

Obesity has become a global public health challenge, elevating itself to the rank of pandemic [1,2]. High Income Countries (HICs) were the first to experience this phenomenon. More recently, Low and Middle Income Countries (LMICs) have been facing a similar, but faster, nutrition transition process [3], so that in Africa, the prevalence of obesity may exceed that found in some HICs [4,5,6]. This phenomenon of nutrition transition is due to changing dietary habits and sedentary lifestyles [7] related to a rapid development of urban industrial environments involving processed high-calorie food, easy access to transports, sedentary occupational and leisure time activities at home [8]. These urban components make the modern city as a built environment physically obesogenic [9], and therefore associated with increasing rates of non-communicable diseases (NCDs), such as hypertension and type 2 diabetes [10].

In addition, the external migration to HICs of these populations has consequences for public health. Indeed, the length of stay of migrants in urban environments in HICs could be associated with the development of obesity and related NCDs [11,12]. In recent history in HICs, obesity has mainly affected women [13] and lower socio-economic groups [2], particularly those living in urban areas, even though it now affects all socio-economic groups [14]. The reasons for this are complex, but include the trend to consume a cheaper and more monotonous high-calorie diet [14,15,16]. Indeed, besides a greater accessibility of this food to working-classes living in poor urban neighbourhoods, some authors provided a cultural explanation: the phenomenon of “social revenge”. For these less educated populations who experienced food shortages in their past, this includes the desire to discover “the pleasures of the city”, including “good food” and weight gain [17].

African migrants living in HICs are more vulnerable to socio-economic insecurity, and to higher morbidity and mortality from NCDs [18]. In addition, they bring with them from Africa a socio-cultural preference for stoutness, the lay representation of overweight [19], that is not always adapted to the urban ecosystem [20]. This propensity for valuing stoutness takes root during internal migration in African countries, where newly arrived migrant groups envision the modern African city as a resource-rich environment, and thus view stoutness as an indicator of their successful integration into a modern urban lifestyle. If social valorisation of stoutness takes root in rural areas where food resources are scarcer, this preference is limited since a strong physical body is necessary for manual labour [21]. This sociocultural phenomenon becomes pronounced and therefore a maladaptation in obesogenic urban areas, where new migrants persist in the belief that fat reserves are advantageous, stemming from the advantage against seasonal adversity this confers in rural areas. Urban migrants are therefore proud to expose their overweight status in an ostentatious way [20,22], so that this cultural preference for stoutness can be considered as a risk factor for overweight [23,24] involving high-calorie eating practices and low physical activity [25,26].

Furthermore, despite the cult of thinness in Western countries [27], this valorisation of stoutness may persist amongst poor African migrant populations living in HIC cities [18]. Thus, the few studies conducted among African minorities in France suggest that obesity is ultimately a threat to life expectancy of first-generation migrants, particularly for African women and their unborn children [28]. The district of Seine Saint-Denis, a working-class area of outer Paris with a high density of immigrant populations, has a prevalence of obesity of 17.1% [29] which is higher than the inner Paris [11,30]. To explain this, de Capèle et al. highlighted the social valorisation of high-calorie food as a crucial determinant of this trend [29]. For example, the high-calorie Malian diet would involve culinary practices that include consuming high quantities of cooking oil, symbol of prosperity in the household [26,31]. Added to this is the consumption of fast food, as eating hamburgers is seen as a way to “Westernise” oneself in low income immigrants [29].

The role of sociocultural factors in the development of obesity has been demonstrated in Africa [32], particularly in Cameroon. If this country has a high prevalence of urban obesity with the development of obesogenic components of urban industrial environment [33], this phenomenon could be compounded by a pronounced social valorisation of stoutness promoting the desire to deliberately gain weight [19]. This especially concerns the native population of West Cameroon, the Bamiléké, one of Cameroon’s major ethnic groups with around three million inhabitants. This population has high rates of overweight in rural areas, leading to high rates of obesity in Yaoundé [34]. Indeed, many anthropological factors related to the Bamiléké’s lifestyle [19] are potentially obesogenic such as fattening practices, a traditional diet mainly based on palm oil, massive rural exodus and economic success in Cameroonian cities. The Bamiléké in Yaoundé had a significantly higher birth-weight than other ethnic groups [35] and their risk for obesity outweighs that of others in Cameroon by a factor three [34]. During the last 30 years, the Bamiléké who have left Cameroon have mainly migrated to the poor suburbs of the Paris region, France [36], taking with them perceptions of food and stoutness that are ill-adapted to this obesogenic environment [37].

Since Bamiléké lifestyle is influenced by both physical and sociocultural obesogenic environments, we conducted an anthropological investigation on obesity in this migrant population to identify how biological and sociocultural determinants articulate with each other to expose Bamiléké migrants to obesity (Figure S1). This study aimed at examining the biocultural determinants of obesity [38]: stoutness valorisation, sedentary behaviours and high-calorie food, in a dynamic social-ecological context [8] of rural-to-urban migrations of the Bamiléké to Cameroon (Yaoundé) and to France (Paris region).

## 2. Materials and Methods

### 2.1. Study Design

We conducted a mixed methods anthropological study among Bamiléké population (subjects had at least one Bamiléké parent and perceived themselves as Bamiléké) from rural Cameroon (West region), urban Cameroon (Yaoundé) and France (Paris region). This cross-sectional study was based on sociocultural qualitative and quantitative data collected by semi-structured interviews and a questionnaire, including a Body Size Scale (BSS) associated with a Body Image Assessment Guide (BIAG) to identify behavioural determinants of overweight, with biological data collected by anthropometric measurements to assess respondents’ nutritional status. Using data triangulation, we explored the biocultural determinants of obesity qualitatively, which were then tested quantitatively, to evaluate their differential associations with nutritional status in both internal (West rural Cameroon to urban Yaoundé) and external (Cameroon to Paris) migrants. Thus, we used a combination of qualitative and quantitative approaches to: (i) investigate the biocultural relationship between diet, physical activity, body weight norms and obesity development; and (ii) assess how these phenomena impact on a specific population in three different socio-ecological areas (Table 1).

### 2.2. Qualitative Study

#### 2.2.1. Study Process

This study investigated qualitatively the biocultural determinants of overweight by: (i) describing the different vernacular perceptions associated with body weight, i.e., fertility, power, well-being, good health and beauty [39]; (ii) identifying the different local culinary practices that characterize the Bamiléké diet; (iii) establishing the relationship with physical activity; and (iv) exploring if a modern lifestyle through acculturation is linked to a thinness valorisation [40] and influence the determinants of obesity. The qualitative study was instrumental in the development of the quantitative study, since the variables tested by our measurements tools (BSS, BIAG and an adapted food frequency questionnaire (FFQ)) cannot be isolated from their socio-cultural context. Thus, we used the results of this qualitative study to: (i) identify Bamiléké’ norms of diet, physical activity practices and body weight with greater depth than in a quantitative study; (ii) help construct the culturally appropriate FFQ; and (iii) interpret the findings of the quantitative study.

Hence, we used qualitative data concerning the norms of diet, physical activity practices and body weight, to test for causal links between these three aspects and weight gain, which would have been impossible to establish from cross-sectional quantitative data alone. Thus, we used the combination of qualitative and quantitative approaches to: (i) demonstrate the relationship between dietary intake, physical activity, body weight perceptions and obesity status; and (ii) assess how these phenomena are impacting in this specific population. Data collection was conducted by three authors of the team specialized in sociocultural anthropology: Nicolas Monteillet for rural Cameroon, Nicolas Monteillet and Emmanuel Cohen for urban Cameroon and Nicolas Monteillet for France.

#### 2.2.2. Sampling

We recruited a sample of 12 adults of two different age groups: younger (18–30) years and older adults (50–65) years from various neighbourhoods in each of three investigation areas (Table 1). We chose these two age groups to increase the likelihood to recruit participants who are single without children (18–30) years, and participants who are married and have children (50–65) years. We conducted semi-structured interviews according to the standard procedure for qualitative studies. Indeed, this method provides a great freedom of expression to interviewees and develop an interview guide with a malleable frame according to the responses of each interviewee [41]. In Cameroon, we selected participants inside a network of relationships from a local interviewer with experience of conducting local anthropological studies. This network was wide since it was based on relationships of casual acquaintance with our local interviewer; and these relationships were across different neighbourhoods, which meant that new informants could be identified and the initial network expanded. In France, we investigated a large network of Bamiléké through associations of migrants based in the region of Paris. The vast majority of first or second generation migrants are aware of the existence of these associations. They provide the possibility for new migrants to both integrate into France and also maintain social cohesion and traditions inside the community by organizing meetings, traditional or modern celebrations and marriages. We used information from these Bamiléké community associations to expand our sampling frame and to meet other Bamiléké outside the associations’ networks; for example through typical Bamiléké restaurants and specific Bamiléké neighbourhoods.

Thus, contrary to the quantitative study, participation of subjects was somewhat arbitrary as: (i) their agreement to participate in the detailed protocol may have depended on their inclusion in our network; and (ii) our study required participants with sufficient knowledge and interest regarding the study, i.e., the local perceptions of body size, diet and physical activity. These two criteria were simultaneously assessed while screening candidates to participate in the study.

The final sample had a balanced sex ratio. Individual interviews were performed at home or inside Associations’ centres, to allow participants to express themselves in a familiar context without exterior pressure, and were recorded with a handheld recorder. Anthropological studies commonly base their prospective tools on vernacular language to precisely understand local cultural norms. Bamiléké living in Cameroon and France come from different sub-groups and speak different dialects; even if they traditionally share many cultural similarities (e.g., funeral traditions and food habits), they do not fully understand each other’s language and cultures. Therefore, the semi-structured interviews (and all other methods applied in this study) were conducted in French, their vehicular language, and directly transcribed in French. The verbatim cited in this study were directly selected from the original transcription of interviews. In Yaoundé, oral French literacy of interviewees was generally good enough to conduct the interviews in French. In rural areas and in few cases in Yaoundé—mainly among the elders—a young adult in the neighbourhood performed the oral translation of the interview where required.

### 2.3. Quantitative Survey

#### 2.3.1. Study Process

This study investigated quantitatively the associations between the biocultural determinants of overweight identified in the qualitative study and participants’ obesity status. Quantitative analyses allowed us to assess in large scale the level and strength of the association between the potential valorisation of stoutness, high-calorie food consumption, physical activity level and nutritional status, in the context of migration to urban areas of a specific population.

#### 2.3.2. Sampling

In the three settings, we used a two-stage sampling strategy. First, we selected potential sites where adult Bamiléké (≥18 years old) lived or gathered, by randomly choosing in an exhaustive list of Bamiléké villages for rural participants, of neighbourhoods for urban participants in Yaoundé, associations and restaurants for urban participants in the Paris region (Table 1). We recruited study participants in: one village in rural area, three urban neighbourhoods in Yaoundé, three migrant associations and three restaurants in the Paris region (1st degree). Second, inside these respective clusters, we randomly selected one of every three persons for inclusion (2nd degree). We excluded pregnant women and infirm elderly to collect reliable biological and cultural data.

#### 2.3.3. Perceptions of Corpulence

**Figure 1:**
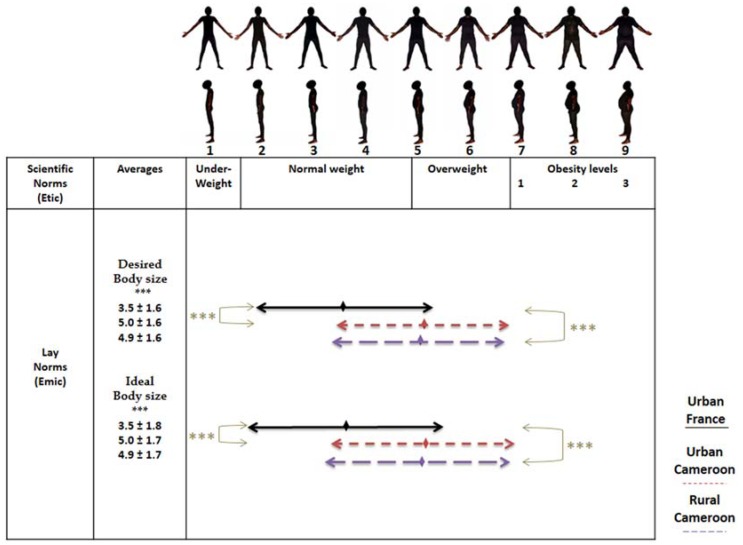
Perceptions of desired and ideal body sizes on masculine Body Size Scale (BSS). Adjusted for age. *** *p* < 0.001.

**Figure 2:**
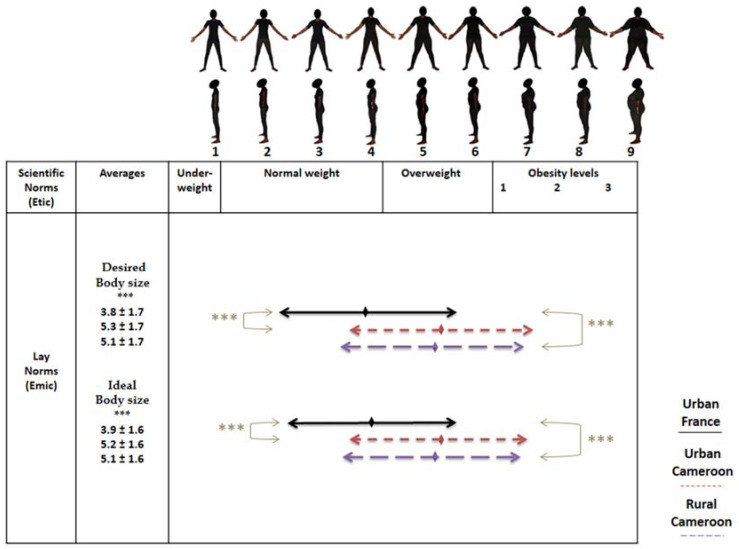
Perceptions of desired and ideal body sizes on feminine BSS. Adjusted for age. *** *p* < 0.001.

To accurately assess body perceptions, we used the validated BSS (Figure 1 and Figure 2) [42]. Then, we built a BIAG to contrast local Bamiléké norms with scientific norms of body weight measured by the BSS. The BIAG consists of four questions about desired body size (DBS) and ideal body size (IBS) for oneself [43], as well as one’s partner [44], to assess possible differences in corpulence norms in contrasting geographical settings. We also used two questionnaire items to assess the social valorisation of stoutness [32]. The first question was: “Do you want to gain weight, lose weight or stay the same size?” The second question was: “What does overweight symbolize for you?” The possible answers were: “1. Wealth, 2. Respectability, 3. Disease, 4. Difficulties”. The first two modalities were coded in “stoutness valorisation” and the last two in “stoutness depreciation”.

#### 2.3.4. Food Frequency

**Figure 3:**
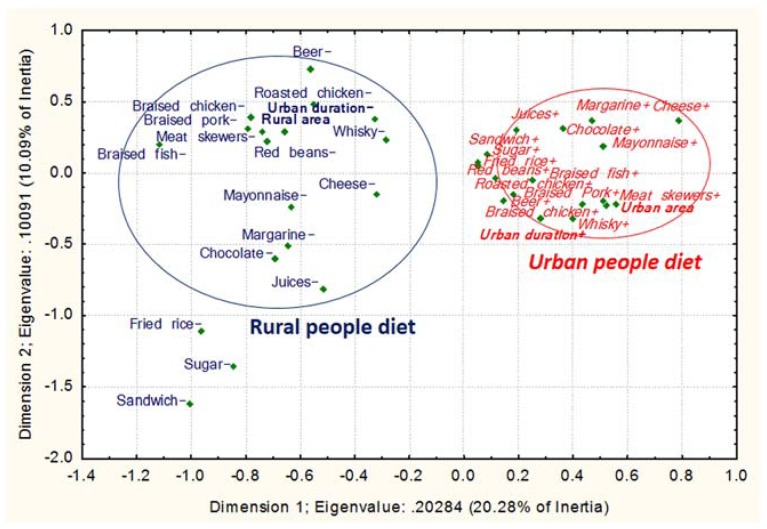
Modern high-calorie foods/dishes depending on migration profile in Cameroon. Multiple Factorial Correspondence Analysis (MCA) 1 between high-calorie foods and two migration variables. The food variables on the left hand side followed by the “−“ sign are consumed less than those in italics on the right hand side followed by the “+” sign. The variables in italic and normal text represent the two patterns of food consumption according to the migration status. The variables in bold represent the non-food variables. The encircled variables were significantly associated with migration status.

**Figure 4:**
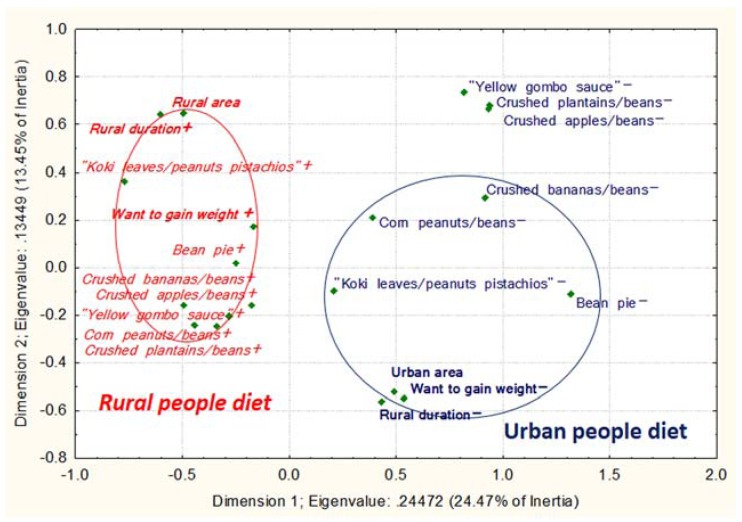
Traditional high-calorie foods/dishes depending on migration profile and corpulence perception in Cameroon. MCA 2 between high-calorie foods, two migration variables and one corpulence perception variable. The food variables on the right hand side followed by the “−” sign are consumed less than those in italics on the left hand side followed by the “+” sign. For the corpulence perception variable, “+” means the desire to gain weight and “−“ the desire to maintain or lose weight. The variables in italic and normal text represent the two patterns of food consumption according to the migration status. The variables in bold represent the non-food variables. The encircled variables were significantly associated with migration status.

**Figure 5:**
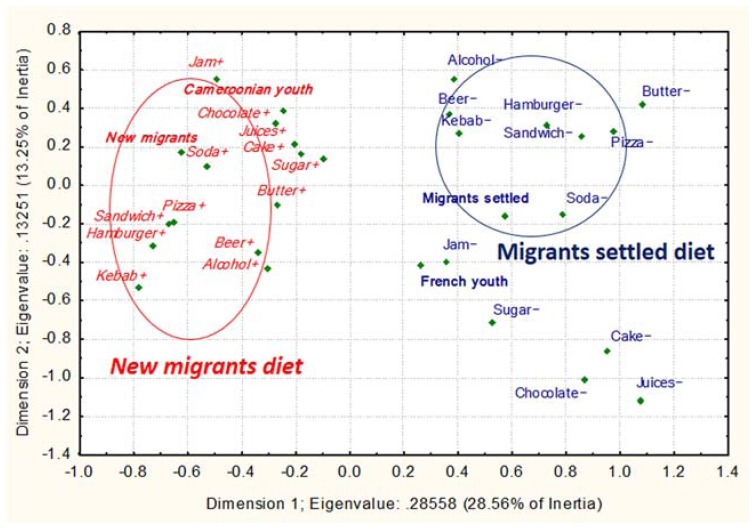
Modern high-calorie foods/dishes depending on migration profile in France. MCA 3 between high-calorie foods and two migration variables. The food variables on the right hand side followed by the “−“ sign are consumed less than those in italics on the left hand side followed by the “+” sign. The variables in italic and normal text represent the two patterns of food consumption according to the migration status. The variables in bold represent the non-food variables. The encircled variables were significantly associated with migration status.

**Figure 6:**
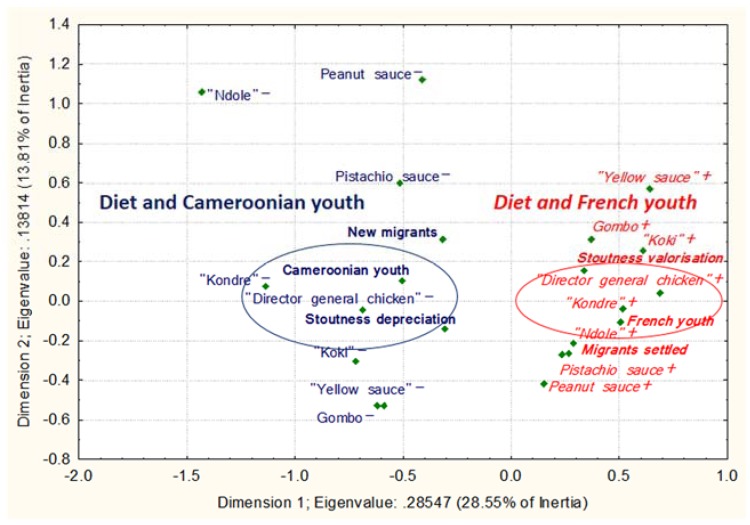
Traditional high-calorie foods/dishes depending on migration profile and corpulence perception in France. MCA 4 between high-calorie foods, two migration variables and one corpulence perception variable. The food variables on the right hand side followed by the “–” sign are consumed less than those in italics on the left hand side followed by the “+” sign. The variables in italic and normal text represent the two patterns of food consumption according to the migration status. The variables in bold represent the non-food variables. The encircled variables were significantly associated with migration status.

To assess the consumption of energy-dense food, we created a qualitative FFQ incorporating high-calorie food including processed modern food mainly found outside the home and traditional Bamiléké dishes which belong to its vernacular culinary practices based on peanut and palm oils [45] (Figure 3, Figure 4, Figure 5 and Figure 6). The list of foods was constructed from qualitative and quantitative data on Cameroonian dietary habits collected by authors from our team (Norbert Amougou, Emmanuel Cohen, Nicolas Monteillet, Rihlat Saïd-Mohamed, and Téodyl Nkuintchua) in this study and a previous study which aimed at identifying main local dishes/foods to assess Cameroonian food consumption [46]. We were able to construct a culturally appropriate FFQ, adapted to local culinary practices, as in other African studies [47]. For each food or meal, we asked respondents to estimate their frequency of consumption on six modalities: “1. Almost every day or every day, 2. Three to five times per weeks, 3. Once or twice per week, 4. Twice to three times per month, 5. Once per month or less, 6. never”. The first four modalities were coded in “consumed regularly” or “food+”, and the last two in “low consumption” or “food−“. We chose this categorisation since daily, weekly and frequent monthly consumptions of these high-calorie foods could strongly higher expose to cardiovascular and metabolic diseases than consuming these food items only a few times a year or less.

#### 2.3.5. Physical Activity

To define and assess the level of physical activity, we used two items from the International Physical Activity Questionnaire (IPAQ) [48]: the intensive level of physical activity (digging, carrying heavy loads, make efforts intensely, etc.) and the moderate level of physical activity (gentle cycle, carrying light loads, make efforts moderately, etc.). We assumed that this would be the type of physical activity that varied the most between rural and urban areas due to the abandonment of farming and the development of the services economy involving more sedentary occupational activities [49]. The average level of intense and moderate daily physical activity (in hours) were calculated from the number of days and the duration each physical activity levels were reported by the participants for the seven days prior to completing the questionnaire.

#### 2.3.6. Anthropometry and Blood Pressure

A set of anthropometric measurements was taken by the same trained fieldworker, using standardized procedures [50]. Height was measured to the nearest mm using a portable stadiometer (Siber Hegner, Zurich, Switzerland). Weight was measured, in very light clothing, to the nearest 100g, using a digital beam scale (Tanita, Tokyo, Japan). Body Mass Index (BMI) was calculated by dividing weight in kilograms by the square of height (in meters). Obesity was defined from 30 kg/m^2^. Hip and waist circumferences (HC and WC) were measured, to the nearest mm, in a standing position using a non-stretchable tape measure, according to standard procedure. Waist to hip ratio was calculated to assess body fat distribution. In addition, an average of two diastolic and systolic blood pressure (BP) readings were taken with subjects in a seated position, according to standard procedures, after 15 min rest. We defined the blood pressure risk factor at: diastolic BP ≥ 90 and/or systolic BP ≥ 140 [1].

### 2.4. Data Analysis

#### 2.4.1. Qualitative Study

Following the recommendation on the methodologies of investigation in anthropological studies, we adopted an inductive approach without a pre-identification of sociocultural determinants related to obesity in order to understand the local perceptions of this population towards obesity development [41]. Discourse analyses were performed using thematic groupings approach to identify emerging themes for each determinant of obesity [51]. The themes emerging from the interviews were identified from participants’ point of view on biocultural determinants of obesity. After the identification of main themes, we summarised the main information of each theme and compared the social representations and practices related to them between our three subsamples.

#### 2.4.2. Quantitative Study

##### Migration Status

In Cameroon, the migration profile of subjects was deduced through the creation of one variable, the “duration of residence in urban area” coded into two categories: “urban duration-” or “short-lived in Yaoundé”: ≤15 years of residency in Yaoundé (*n* = 265; age = 35.8 ± 14.1); and “urban duration+” or “long life in Yaoundé”: >15 years of residency in Yaoundé (*n* = 309; age = 41.0 ± 12.9). In France, the migration profile was deduced through two variables. The first variable “duration of residence in France” was coded into two categories: “new migrants” or “short-lived in France”: ≤15 years of residency in France (*n* = 24; age = 38.8 ± 6.3); and “settled migrants” or “long life in France”: >15 years of residency in France (*n* = 26; age = 53.5 ± 11.3). The second variable “duration of residence in Cameroon” was coded as: “French youth” or “life time short in Cameroon”: ≤25 years of residency in Cameroon (*n* = 24; age = 46.9 ± 14.8); and “Cameroonian youth” or “long life in Cameroon”: >25 years of residency in Cameroon (*n* = 26; age = 45.9 ± 8.5). We chose these cut-off points because these values are close to the medians of both Cameroonian and French samples.

##### Dietary Intake

A high-calorie diet index was constructed using a Principal Component Analysis (PCA) from the main traditional and modern energy-dense dishes/foods commonly consumed (Tables S1–S3) in the three areas (14 “modern dishes/foods” and 16 “traditional dishes/foods”). All these dishes/foods were considered nutritionally energy-dense since their energy value could expose to obesity and related pathologies if they are regularly consumed. For instance, the consumption three times a day of a medium portion (250 to 700 g) of a common adult meal such as peanuts sauce (101.8 kcal/100 g dry matter) [39], will lead to 764 to 2290 kcal energy intake per day [46,52,53]. This implies that the sole consumption of larger portions is already over the recommended daily energy requirements (2000–2500 kcal/day) [54], not accounting complementary food usually eaten along with peanuts sauce (rice, plantain, drinks, etc.). High-calorie diet was classified into three distinct groups: “lower”, “middle” and “higher” levels according to tertiles of the calculated variable from the first principal component (PC1). In addition, the Multiple Factorial Correspondence Analysis (MCA) of high-calorie traditional and modern dishes/foods consumed was used for the analysis of food consumption in the context of migration (Tables S1 and S2).

##### PCA and MCAs

We performed the PCA, assessed its goodness of fit, and selected principal component following several steps: (1) KMO (Kaiser-Meyer-Olkin) measure; (2) the Bartlett’s test of sphericity on the matrix correlation of food variables; (3) Kaiser’s criterion (eigen value s> 1); (4) scree test (elbow on the scree plot); and (5) factor loadings.

For the MCAs, we assessed the goodness of fit and selected the relevant dimensions using: (1) Cronbach’s alpha for each dimension; (2) Kaiser’s criterion; (3) scree test; and (4) the discrimination measures of food items on each dimension.

##### Body Image

BSS was treated as a numerical variable, as each human picture ranged from 1 to 9 according to increasing BMI categories. Using the BSS, a social valorisation of stoutness index was constructed with this formula: DBS − 4; since the fifth silhouette on the scale corresponds to overweight. Thus, a DBS score <4 (desire to be underweight or normal weight) results in negative values of the social valorisation of stoutness index which means a non-valorisation of stoutness. A DBS score ≥4 (desire to be overweight or obese) results in zero or positive values which means a valorisation of stoutness.

##### Physical Activity

A physical activity index was constructed from the two IPAQ items by averaging the time spent weekly in intense and moderate physical activity (hours/week).

##### Relationships between Beliefs, Practices and Body Weight

With a binary logistic regression model in the total sample, the relationships (adjusted odds ratios, 95% confidence intervals) between obesity and their possible determinants (sex, age, living area, educational level, high-calorie diet index, social valorisation of stoutness index, and physical activity) were assessed. ANOVA, ANCOVA, Student test and chi^2^ were used to compare anthropometric measures, body size perception averages, food consumption, physical activity, and prevalence of obesity and hypertension in the total sample.

We used NVivo 7 software (QSR International, Melbourne, Australia) to summarise the main outcomes of the interviews. We used the Statistica 7 (Statsoft Inc., Tulsa, OK, USA) and IBM-SPSS 21 (IBM Co., Armonk, NY, USA) softwares to perform descriptive statistics, ANOVA/ANCOVA, MCAs and PCA, and My Stat 12 software (Systat Software Inc., San Jose, CA, USA) for the logistic regression. Finally, qualitative and quantitative approaches were assigned equal weight in the interpretation of the findings.

### 2.5. Ethics

This study was conducted according to the guidelines laid down in the Helsinki Declaration and all procedures followed were approved by the Institutional Ethics Committee of the Institute of Medical Research and Medicinal Plant Studies of Cameroon. Oral and written consents were obtained from subjects, after being fully informed of the study goals and methods.

## 3. Results

### 3.1. Qualitative Study

#### 3.1.1. The Social Perceptions of Stoutness

Bamiléké from the village setting already had a strong social valorisation of stoutness. Different initiation rituals legitimise the prestigious status of notables (wealthy/high ranking people) and the king, especially by their development of stoutness. Before the future king is crowned, he has to live shut away in a special house for two months, known as the lacam, to reach a certain weight. Notables also need to gain weight with age “to fill” their large traditional clothes, njocho.

- Man, rural area, king, thirties:
  “In principle, only notables can wear this kind of clothes, the njocho, it means: “the clothes of titan”. In lacam, after nine weeks of confinement, you have to be like this (very large corpulence). Every day, you must eat palm oil, they even anoint you with it. Concerning my weight, it made me change. Being well means being with your belly, the administrative belly. The Chief’s belly announces fertility for the village in the near future.”

We can observe the same phenomenon for women, the ritual is called mezin. They have to gain weight to be considered as fertile, beautiful, comforting in the home, therefore ready for marriage.

- Man, rural area, notable/farmer, sixties:
  “Some women before marriage were undergoing an initiation ritual, the mezin. Women who cannot easily grow, too feeble, they were in competition for the mezin path. They ate to become fat. Once they enter mezin, they embalm themselves with palm oil as makeup. During two or three weeks, they bathe in oil, they eat a lot, nkui couscous, pistachio sauce, tarot yellow sauce, they coddle. Afterwards their corpulence has changed, they become plump. They gain respect after that, they are pretty. For a while, they do even more work in the fields.”

These rural perceptions of weight persist on migration to the city, Yaoundé. Indeed, weight becomes a symbol of economic success for men, which is commonly called the “administrative belly”.

- Man, urban area, pupil, twenties:
  “I want to get to 70 kilos (1.63 m; 67.1 kg). When I will have more money, I want a belly. When you see the belly, here’s a boss! When the man has money and he hasn’t got a belly, people say that he does nothing with it.”

In an urban context, where socioeconomic integration to the city is not easy, after marriage, the wife’s weight gain is a symbol of the capacity of the husband to manage his family.

- Woman, urban Area, student, twenties:
  “For a woman, being overweight suggests that her husband takes good care of her, that he is comfortable, he has money.”

Nevertheless, with external migration to France, body size perceptions change. BMI preferences are more assimilated into local norms, whereas traditional norms are questioned, so that stoutness is less associated with well-being and the health benefits of engaging in physical activity are acknowledged.

- Man, Paris area, night watchman, thirties:
  “I enrolled in a sports club yesterday for a year. So, the goal is to maintain fitness and refine a little bit my body. I know that doing sport over a year, I will lose at least four or five kilograms. But after that, I can no longer lose. Sports must be regular, at least two three times a week. I have a BMI of 25.5. Normal is between 18 and 25. Between 25 and 30, one is overweight, between 30 and 35, one is obese, etc. Here I have a BMI of 25.5. My BMI was even higher before the diet. Now, doing sport, I bring it down below 25. I know that in Africa, when you’re like I am, that’s fine. At home (in Cameroon), you are told: “If it is not the food that kills someone, it will be something else”. In Cameroon, I have even heard people telling me that I need to drink more beer because I do not have enough belly”.

#### 3.1.2. Diet Perceptions and Practices

Bamiléké people have abundant food resources in West Cameroon, with culinary practices based on regular palm oil consumption, present in large quantities in most traditional meals.

- Man, rural area, notable/farmer, sixties:
  “In traditional food, we eat taro, yams, corn couscous, but rarely fruits. In our tradition, we ate what was particularly good. Fruit like guava are food for children. A man of my age cannot eat it. But we ate vegetables; moreover, some of our traditional vegetables, they are not eaten today. At that time, there were no shortages, with agriculture, there was no famine. Historically, in the village, I have never known people who were hungry. Palm oil is the basis of our diet; without palm oil, people would panic. Palm oil is essential, everything is prepared with it. This is a good food, it gives strength everywhere.”

Nevertheless, with the nutrition transition, food has become more processed and energy-dense, and its quality is seen as lower in the city than in the village. In addition, dietary intake is less structured, more individualised, composed of frequent snacking, which is rare in the village setting.

- Man, rural area, king, thirties:
  “The traditional oil is oil-based palm nuts. Today, concerning the industry, it is not the oil of the village anymore, it is not nutritious. So much has changed, it has blown all the vitamins. We bleached oil, after there is no more carotene. Traditionally, we did not fry the oil like that, we also did not fry vegetables constantly, they are boiled. We ate well, there is not even a concept to express “snack” in tradition, we only ate three times a day.”

Parisian Bamiléké migrants observe the same phenomenon as migrants in Yaoundé concerning the poor quality of urban diets. They believe that processed food exposes populations more to overweight, than traditional diets do.

- Man, Paris area, manager, thirties:
  “Since we introduced frozen chicken at home, things like that, and people eat because they think it’s quick, they directly have the “Western diseases”, those did not exist before, such as diabetes. All this is due to modern food. I have many friends, when I visit them at home, it is only modern food. So they are big, they are potbellied, there is the belly, they abnormally gain weight while I’m still frail. I see the difference. The food from the home country gives strength to the body, but it is not fattening because in fact it is healthy. In fact, there are less unnatural things in the diet that will be added to the body to swell it.”

Nevertheless, even if most Parisian Bamiléké migrants want to keep their traditional dietary habits, economic accessibility makes it difficult.

- Woman, Paris area, housewife, fifties:
  “It bothers me! But fortunately, I started with my children from an early age, already little, giving them Cameroonian dishes. I am Cameroonian, so: “You need to eat Cameroonian dishes". They began to eat that a little, so that when they went to Cameroon, they do not really discover anything.”
- Man, Paris area, Paris area, handler, thirties:
  “Here, the real problem with food from the home country is the price. Home country foods are expensive. Frozen costs less.”

#### 3.1.3. Physical Activity

Bamiléké people attest that intense physical activity is/was frequent as a consequence of a rural lifestyle since most people are/were farmers. These subsistence activities imply good fitness which prevents/prevented massive weight gain. Nevertheless, the urban lifestyle in Cameroon as well as in France means that these ecological factors change, with morbid consequences on the body.

- Woman, Paris area, housewife, sixties:
  “In fact, as we are too sedentary in our country, we do not practice sports. I’m talking about moms who are now in town, because our moms were physically active. They worked in the fields. So they did not have all these problems. But after people moved into town, the settlement and all that...”

Parisian Bamiléké migrants try to adapt their social lifestyle to these ecological conditions but it is difficult to adopt a daily level of physical activity similar to that in the village of origin, since this activity is not as necessary in urban areas, and the busy schedule caused by modern lifestyles does not leave enough time for hobbies.

- Woman, Paris area, housewife, sixties:
  “When I can, I walk. I would not take my car to buy bread as some people do. Sometimes, I go to the same post office five times a day by foot. I try to avoid the damage (of a sedentary lifestyle). There are some moments when we can do this. In winter, you cannot. The weather now is sad. At 3pm, it is already dark, we do not have the courage to go out.”
- Man, Paris area, handler, thirties:
  “I do not run anymore because there is not really enough time. Time is saturated by work at the store.”

### 3.2. Quantitative Survey

#### 3.2.1. Perceptions of Corpulence

We firstly observed that for both masculine and feminine scales, averages of DBS and IBS were significantly lower in Parisian Bamiléké than in rural and urban Cameroonians. Secondly, there were no differences in DBS or IBS between rural and urban Cameroonians (Figure 1 and Figure 2). On the masculine scale (Figure 1), overall, Parisian Bamiléké had a DBS in a healthy “normal” BMI category, whereas rural and urban Bamiléké desired a body between normal-weight and overweight categories. We found the same pattern of results for IBS.

On the feminine scale (Figure 2), we observed similar trends. Nevertheless, rural and urban Cameroonians have both DBS and IBS in the overweight category.

#### 3.2.2. Dietary Intake

For MCAs 1 to 4 (Figure 3, Figure 4, Figure 5 and Figure 6), (1) Cronbach’s alpha were acceptable to good, and the highest for each first dimension (D1): 0.77, 0.66, 0.82 and 0.77, respectively. Each D1 met the (2) Kaiser’s criterion and (3) scree test, and had (4) the highest discrimination measures with most items (Tables S4–S7). The second dimension of each MCA presented the second highest Cronbach’s alpha and global discrimination measures but our interpretation was based on the D1s, the only ones presenting acceptable Cronbach’s alpha.

To investigate the frequency of consumption of high-calorie “modern” foods (e.g., meats, cheese, chocolate, sodas, juice and alcohol; see Table S1), we conducted a MCA in rural and urban Bamiléké in Cameroon (MCA 1, Figure 3). The results showed that life in an urban area, especially >15 years of residency, was associated with more frequent consumption of high-calorie modern foods, whereas life in a rural area and ≤15 years of residency in an urban area, was associated with lower consumption of these high-calorie foods (*p* < 0.001; *n* = 568).

The MCA 2 (Figure 4) was conducted to synthesize the frequency of consumption of high-calorie traditional Cameroonian foods and dishes (e.g., “pistachio sauce”, “ndolé”; see Table S1). We observed that an urban lifestyle was associated with lower consumption of high-calorie traditional foods and a rural lifestyle was associated with more frequent consumption of these traditional foods and a desire to gain weight (*p* < 0.001; *n* = 573).

In the Parisian sample, we conducted the MCA 3 (Figure 5) to synthesize the frequency of consumption of high-calorie modern foods (Table S2). Recent arrival in France, with a longer lifetime spent in Cameroon, was associated with more frequent consumption of high-calorie modern foods, whereas arrival in France a long time ago, with a short lifetime in Cameroon, was associated with lower consumption of this modern food (*p* < 0.001; *n* = 50).

Then, we conducted the MCA 4 (Figure 6) of the frequency of consumption of high-calorie traditional Cameroonian foods and dishes (Table S2). Recent arrival in France, with a long lifetime in Cameroon, was associated with lower consumption of high-calorie traditional foods, whereas arrival in France a long time ago, with a short stay in Cameroon, was associated with more frequent consumption of traditional foods (*p* < 0.001; *n* = 50) and a positive body fat perception symbol of wealth and respectability.

In both countries, the high-calorie traditional and modern foods/dishes included in these analyses represented a large part of high-calorie foods/dishes regularly consumed by Cameroonians. In addition, analyses on the traditional food showed that the difference between the two patterns of food consumption of Cameroonian migrants was greater when a variable on body weight perception was added. Then, the associations between foods/dishes and migration status (Tables S1 and S2) found significant in univariate analyses were circled highlighting the opposite diet patterns related to each group investigated in the two countries.

### 3.3. Physical Activity

When adjusted for age, level of intense physical activity was higher in rural Cameroonian (2.6 ± 2.1 h) than those in Cameroonian and French urban groups (0.8 ± 2.1 h and 0.07 ± 2.1 h, *p* < 0.001), and in those living in urban Cameroon compared to Paris (*p* < 0.01). Similarly, the level of moderate physical activity was higher in rural Cameroonians (3.4 ± 2.2 h) than those in urban Cameroon or France (2.9 ± 2.2 h and 0.4 ± 2.2 h, *p* < 0.05 and *p* < 0.001), and in those living in urban Cameroon compared to Paris (*p* < 0.001).

### 3.4. Migration Status and Determinants of Obesity

We observed that the residence duration in Yaoundé was significantly associated with a more frequent consumption of a higher-calorie diet and lower moderate and intense physical activity, but not with DBS (Table 2). The residence duration in the Paris region was not associated with these determinants, even if we observed that a frequent consumption of a higher-calorie diet was higher with the recent migration.

### 3.5. Anthropometry and Blood Pressure

Women were fatter and had lower blood pressure than men. In both sexes, individuals from urban areas had larger anthropometric indexes of adiposity and higher diastolic blood pressure than those from rural Cameroon. We also observed in women that there were no significant differences of BMI, WC and HC between urban Cameroon and France (Table 3).

Systolic blood pressure was significantly higher in urban than in rural Cameroon in women. In addition, the prevalence of obesity and elevated blood pressure were significantly higher (*p* < 0.001; *p* < 0.05) in urban areas. For obesity, we found a prevalence of 13.2% in rural Cameroon (*n* = 34), 32.0% in urban Cameroon (*n* = 102), 40.1% in urban France (*n* = 20). For elevated blood pressure, we found a prevalence of 22.2% in rural Cameroon (*n* = 57), 27.0% in urban Cameroon (*n* = 86), 40.1% in urban France (*n* = 20). Finally, a significant association was observed between obesity and hypertension in the total sample (*p* < 0.001).

### 3.6. Analysis of Obesity Determinants

From the PCA on the food variables, we extracted the factor explaining the largest variance and inserted it as a proxy for high-calorie food intake in the logistic regression (Table 4). On the matrix correlation, the (1) KMO measure was good (0.79) and (2) the Bartlett’s test of sphericity was significant (*p* < 0.001). PC1 met the (3) Kaiser’s criterion and (4) scree test, and explained 14.3% of the variance (eigenvalue of 4.3, and 2.4 for the second principal component). It had the (5) highest factor loadings (Table S8): all correlations with food variables were in the same direction, 15 of them had correlations higher than 0.30, plus five with a correlation >0.28, which led us to retain this first component.

The comparison of obese (≥30 kg/m^2^) with non-obese subjects revealed an independent association of sex, age, migration status and valorisation of overweight (or obesity). These factors were independently associated with obesity status. Obesity status was more frequent in women than in men and positively associated with age, urban residence duration, lower physical activity and valorisation of overweight.

## 4. Discussion

This study investigated the biocultural determinants of obesity (stoutness valorisation, eating and physical activity practices) among Bamiléké people in the dynamic social-ecological context of rural-to-urban migration in Cameroon (Yaoundé) and to a French urban environment (Paris). Overall, we observed that this population has experienced health effects from both internal and external migration to urban Cameroon and France.

Indeed, the Cameroonian population which has settled in Yaoundé and the Paris region [37] has high rates of obesity with biocultural determinants acting in different ways to be associated with the spread of obesity from one area to another, as has been observed in other migrant populations in urban areas of LMICs and HICs [18,55,56,57]. The nutrition transition in this population is in an advanced stage, particularly in women who had similar prevalence of obesity in both urban Cameroon and France. Our results suggest that women have already reached a stage of the nutrition transition in urban Cameroon—where prevalence of obesity is high—comparable with the prevalence of women living in HICs [58]. One explanation is that Cameroonian women are more exposed to the obesogenic effects of an urban lifestyle [34], as in other LMICs [59].

Firstly, according to our qualitative analysis, despite the social valorisation of overweight that is pronounced in Bamiléké culture, it seems to fall with external migration to Paris. Parisian Bamiléké seem to adapt their body size norms to those of the host population, like many other migrant populations living in HICs [60,61,62]. In contrast, the valorisation of overweight in Yaoundé is marked and an independent risk factor for obesity. This is in accordance with the high prevalence of obesity (32.0%) and its association with hypertension found in the study, which is similar to previous survey [34]. Migration from West Cameroon to Yaoundé sees the transfer of both: (i) the traditional ritual of the corpulent male ruler after the fattening ritual in the lacam [45] to the modern ritual of the household head with his “administrative belly” [63] acquired through his high professional status; and (ii) the traditional ritual of the fat mezin woman [64] nurturing, soothing and idle with age in marriage, to the modern ritual of an obese housewife expressing the economic success of her husband through her corpulence [65], which sheds light on the reasons behind the quantitative results.

Secondly, the Bamiléké have traditional culinary habits which promote abundant and high-calorie diet also observed from nutrient level analysis [66]. This diet could expose them to overweight in both rural Cameroon and Yaoundé, even if our interviewees suggested that their traditional diet prevents NCDs [33], in accordance with the low prevalence of morbid overweight found in West Cameroon [34]. Indeed, according to our qualitative interviews, the traditional diet in rural Bamileke is based on natural palm oil and unprocessed foods according to our qualitative study, contrasting with the urban Bamiléké’s diet which integrate modern high-calorie and processed foods. With external migration to France, first-generation Parisian Bamiléké migrants maintain, more for economic reasons, a similar diet to those from urban Cameroon, mainly based on fast and processed foods. A similar pattern was observed by Renzaho and Burns [67] in their study of Africans migrants in Australia and by Tuomainen [68] studying Ghanaian migrants living in London.

On the other hand, for migrants that are socio-economically better integrated into the host country’s lifestyle, we observe a phenomenon of “back acculturation” [69]; i.e., settled migrants rediscovering their traditional culture, especially through their diet by re-claiming traditional dishes [68], with more unprocessed foods [70]. Indeed, our interviews reveals that traditional dishes are too expensive for first-generation migrants who spent their youth in Cameroon and who are keen to adapt to the French lifestyle [40]. Eating Cameroonian food represents family cohesion, but requires a substantial socioeconomic status barely accessible to young single migrants, whose status does not allow the adoption of this lifestyle, whereas this is important for middle-aged migrants who want to transmit traditional values to the second generation [70].

Nevertheless, our findings suggest that a frequent consumption of high-calorie foods, whether traditional or modern, does not constitute a risk factor for obesity as previously observed in Benin [71]. Even though we adapted our food frequency questionnaire to the Bamiléké culinary habits [46], this results might result from common bias (recall bias, averaging food consumption) of food frequency questionnaires [72]. A quantitative assessment of dietary intake with a 24-h recall guide containing the respective energy value of each food would provide a more accurate quantification of the nutritional composition of the diet and would allow a better assessment of the relationship between dietary intake and the risks of obesity. However, there are no nutritional composition tables integrating Cameroonian foods/dishes yet. In addition, it would have been interesting to assess metabolic syndrome components (HDL cholesterol, triglycerides, fasting glucose and insulin resistance) to evaluate the relationship between high-calorie diet and obesity-related diseases.

Besides these two aspects, the main limitation of this work is the small sample size in Paris resulting in a decreased statistical power of site-specific analyses. Indeed, the sample size varied between Parisian and Cameroonian Bamiléké because it was harder to access Bamiléké living in the Paris region as they represent only several thousand people [73] and electoral registers do not permit the identification of ethnicity. Nevertheless, the inclusion of this sample allowed carrying out a preliminary comparative assessment of Bamiléké health status as a consequence of external migration [74] and obtained an overview, rarely studied phenomenon among African minorities in France. Governments of HICs are currently faced with the challenge of integration difficulties and precarious living conditions of some immigrant populations, which requires support by public authorities [18], as we can observe for the Parisian Bamiléké. As further limitation, although the two-stage sampling was carefully defined, this clustering strategy could involve heterogeneity between clusters, and homogeneity within each one, which could affect variance estimates in our statistical analyses.

As far as physical activity is concerned, the lower intense and moderate physical activities observed in urban areas were associated with obesity, a tendency reported by Sodjinou et al. [71] in Cotonou in Benin where higher physical activity levels were associated with a significantly lower BMI. Rural Bamiléké have significant agricultural activity according to different studies [45] which certainly offsets a traditional high-calorie diet. However, migration to Yaoundé and France means that this daily intense activity related to work is not replaced with other regular physical activity. Respondents in France reported that their lifestyle (long sitting working hours, caring for children without support other than their partner, and poor weather) is physically incompatible with regular physical activity [75]. In addition, the valorisation of stoutness in urban Cameroon could drive this decrease by the promotion of sedentary behaviours and idleness. This is especially practiced by older married women, who may be proud to delegate, when affordable, household tasks to housekeepers, symbol of peaceful and economic success of the household [25].

Accordingly, as for many African minorities in HICs, Bamiléké who have experienced the migration process may have greater risk of developing obesity [18], even if they are receptive to etic standards (scientific norms) of corpulence in France, questioning the emic (lay norms) valorisation of stoutness [76]. We found that while they can consider a good Bamiléké “natural meal” calorie-rich and invigorating for the body [1], they however value moderate stoutness and fatness rather than overweight. In addition, young acculturated Bamiléké could be torn between two ambivalent models, caught between food hedonism and the valorisation of thinness [77]. These perceptions contrast with those in Cameroon, particularly in rural areas where traditional food is associated with a desire to gain weight and become overweight. However, although Cameroonian urban Bamiléké seem to suffer from obesity due to an over-valorisation of overweight and lower intense physical activity, those living in France have almost totally abandoned any form of physical activity, particularly also moderate physical activity, due to differences in lifestyle. Urban Cameroonians in Yaoundé continue to be active through daily activities such as frequent walking and manual work for men and intensive housework and caring for children/elders for women.

Accordingly, the development of obesity in Bamiléké migrants seems to be due to biocultural factors. We observed: (i) a cultural component in urban Cameroon: the social valorisation of overweight involving sedentary and eating behaviours to deliberately gain weight [31,78]; and (ii) an ecological component in France: lower moderate and intense physical activity related to the development of the transports and comfort inherent to urbanisation process [79,80]. Thus, whilst the Bamiléké of Yaoundé maintain a moderate physical activity, they value inappropriate overweight (in health terms) ill-adapted to the urban ecosystem. In the same way, whilst the Parisian Bamiléké value thinness, they seem to abandon the regular physical activity required for energy balance within an obesogenic environment, particularly those living in poor conditions [15]. Further analysis, with a larger sample in the Paris region, could allow identifying which of these two environments is the more obesogenic in African migrants.

The implications of this study suggest that public health policies need to be adapted for obesity prevention among migrants, accounting for evolving biocultural determinants in different socio-ecological areas. As suggested by Lara, et al. [81], we propose a set of general recommendations in two areas—public health practice and research—targeted to public health personnel in academia, community-based settings, and government agencies since findings from this study could help to promote health initiatives in Cameroonian migrants to link individuals to healthcare services in urban Cameroon and France [82].

## 5. Conclusions

In Cameroon (i), it seems necessary to integrate the valorisation of stoutness as a global determinant of obesity in research protocols and public health nutrition policy, which can expose individuals to unhealthy dietary behaviours [83]. In France (ii), conducting larger epidemiological studies on migrants from LMICs could allow identifying nutritional health outcomes—especially processed and high calorie-foods in working-classes and low physical activity—in the dynamic social-ecological context of external migration and improve public health interventions [84]. To conclude, this study demonstrates that migration can profoundly modify the biocultural determinants of obesity in migrants [85,86]. Therefore, local and global health policies have to consider anthropological specificities of migration pathways to identify the exposure levels of migrants to obesity [87,88].

## Figures and Tables

**Table 1:** Descriptive characteristics of the population samples.

Investigation Areas	Rural Cameroon	Urban Cameroon	Urban France
**Qualitative survey**			
Sample size, *n*	12	12	12
Sex, % men	50	50	50
Age, % (18–30) years	50	50	50
Location	Village of Diambou	Bamiléké neighbourhoods of the capital	Bamiléké migrant associations
**Quantitative Survey**			
Sample size, *n*	258	319	50
Sex, % men	48.8	43.9	56.0
Age, Mean	40.2 ± 15.6	37.3 ± 11.9	46.4 ± 11.8
Location	Village of Diambou	Bamiléké neighbourhoods of the capital	Bamiléké migrant associations. Cultural events: weddings, baptisms, community celebrations. Cameroonian restaurants and at home

**Table 2:** Biocultural determinants of obesity according to the migration status.

Migration Status	Higher-Calorie Diet		Physical Activity		Desired Body Size	
**Cameroonian migrants**						
Yaoundé living duration		*p*		*p*		*p*
0 years	23.2% (*n* = 19)		3.0 ± 1.8 (*n* = 84)		5.1 ± 1.8 (*n* = 83)	
(1–15) years	30.7% (*n* = 55)		2.6 ± 1.6 (*n* = 180)		4.9 ± 1.5 (*n* = 178)	
(16–30) years	42.5% (*n* = 94)		2.1 ± 1.4 (*n* = 222)		5.1 ± 1.7 (*n* = 223)	
>30 years	26.7% (*n* = 23)	**	2.0 ± 1.5 (*n* = 85)	***	5.3 ± 1.7 (*n* = 84)	NS
**Parisian Cameroonian migrants**						
Paris living duration						
(1–15) years	41.6% (*n* = 10)		0.3 ± 0.3 (*n* = 24)		3.6 ± 1.2 (*n* = 24)	
(16–30) years	14.3% (*n* = 2)		0.2 ± 0.3 (*n* = 14)		3.5 ± 1.1 (*n* = 14)	
>30 years	16.7% (*n* = 2)	NS	0.1 ± 0.2 (*n* = 12)	NS	4.1 ± 1.6 (*n* = 12)	NS
Cameroon living duration						
(1–20) years	18.2% (*n* = 2)		0.2 ± 0.3 (*n* = 11)		3.6 ± 0.8 (*n* = 11)	
(21–30) years	27.6% (*n* = 8)		0.2 ± 0.3 (*n* = 29)		3.8 ± 1.3 (*n* = 29)	
>30 years	40.0% (*n* = 4)	NS	0.2 ± 0.3 (*n* = 10)	NS	3.6 ± 1.5 (*n* = 10)	NS

Ancova between the three samples: ** *p* < 0.01; *** *p* < 0.001; NS: No Significant.

**Table 3:** Anthropometric comparisons between Bamiléké according to their living area **^1^**.

Measures	Rural Cameroon	Urban Cameroon	Urban France	
**Men**	*n* = 126	*n* = 140	*n* = 28	*p*
BMI	24.2 ± 3.8 ^c–*c*^	26.9 ± 3.8 ^c–b^	29.4 ± 3.8 *^c^*^–b^	***
Waist circumference (cm)	84.1 ± 9.1 ^c–*c*^	90.3 ± 9.1 ^c–c^	98.8 ± 9.1 *^c^*^–c^	***
Hip circumference (cm)	94.4 ± 8.4 ^c–*c*^	100.6 ± 8.4 ^c–a^	105.5 ± 8.4 *^c^*^–a^	***
Waist to hip ratio	0.9 ± 0.06 ^x–*b*^	0.9 ± 0.06 ^x–b^	0.9 ± 0.06 *^b^*^–b^	**
Systolic Blood Pressure (mmHg)	128.4 ± 19.8 ^x–*x*^	131.1 ± 19.9 ^x–x^	133.0 ± 19.9 *^x^*^–x^	NS
Diastolic Blood Pressure (mmHg)	80.6 ± 13.1 ^b–*a*^	85.2 ± 13.1 ^b–x^	86.6 ± 13.2 *^a^*^–x^	**
**Women**	*n* = 132	*n* = 179	*n* = 22	*p*
BMI	27.0 ± 5.3 ^c–*x*^	29.2 ± 5.3 ^c–x^	28.4 ± 5.4 *^x^*^–x^	**
Waist circumference (cm)	90.1 ± 11.6 ^x–*a*^	91.7 ± 11.7 ^x–x^	96.8 ± 11.8 *^a^*^–x^	*
Hip circumference (cm)	103.4 ± 12.2 ^c–*x*^	108.5 ± 12.2 ^c–x^	107.7± 12.4 *^x^*^–x^	**
Waist to Hip Ratio	0.87 ± 0.06 ^c–*x*^	0.85 ± 0.06 ^c–c^	0.9 ± 0.06 *^x^*^–c^	***
Systolic Blood Pressure (mmHg)	118.5 ± 19.4 ^a–*x*^	123.2 ± 19.5 ^a–x^	117.7 ± 19.8 *^x^*^–x^	NS
Diastolic Blood Pressure (mmHg)	78.4 ± 12.3 ^c–*x*^	83.5 ± 12.4 ^c–x^	80.3 ± 12.6 *^x^*^–x^	**

^1^ Age adjusted by covariance analyses, Ancova between the three samples: * *p* < 0.05; ** *p* < 0.01; *** *p* < 0.001; Post-hoc analyses between the three samples: a < 0.05; b < 0.01; c < 0.001; x: NS for each side-by-side comparison; Superscripts coding: **^a,b,c,x^** Rural Cameroon vs. Urban Cameroon; *^a,b,c,x^* Rural Cameroon vs. Urban France; ^a,b,c,x^ Urban Cameroon vs. Urban France.

**Table 4:** Odds ratio (OR) and 95% confidence intervals (CI) for subjects who are obese, adjusted by binomial logistic regression analysis, and compared to subjects who are not obese in the total sample (*N* = 608).

Predictors	Categories	Obesity	
		*uOR CI*	*aOR CI*
Sex ***	Men ^†^		
	Women	**2.4 (1.6–3.5c)**	**2.5 (1.6–3.8c)**
Age ***		**1.0 (1.0–1.1c)**	**1.0 (1.0–1.1b)**
Migration status ***	No or lower urban living duration ^†^		
	>25 years of Yaoundé residency	**2.5 (1.7–3.8c)**	**1.7 (1.1–2.6a)**
	>10 years of Paris region residency	**3.1 (1.5–6.2b)**	**3.3 (1.2–8.8a)**
Educational level	Others ^†^		
	University	0.7 (0.5–1.2)	0.8 (0.4–1.5)
Calorie-level of diet	Others ^†^		
	Higher calorie diet	1.0 (0.7–1.4)	1.1 (0.7–1.7)
Physical activity ***		**0.8 (0.7–0.9c)**	**0.8 (0.7–0.9b)**
Overweight valorisation ***	Non-valorisation ^†^		
	Valorisation	**3.4 (2.2–5.2c)**	**3.5 (2.1–5.7c)**

*** Crude analysis significant effect (*p* < 0.05; *p* < 0.01 and *p* < 0.001 respectively). In bold, binomial logit analysis significant effects (a, b, c): *p* < 0.05, *p* < 0.01, *p* < 0.001 respectively. ^†^ Category taken as reference. uOR: unadjusted Odd Ratio; aOR: adjusted Odd Ratio.

## Supplementary Materials

The following are available online at www.mdpi.com/1660-4601/14/7/696/s1. Figure S1: Biocultural determinants of obesity among Cameroonian migrants, Table S1: List and consumption frequency of main dishes/foods in Cameroon, Table S2: List and consumption frequency of main high calorie dishes/foods in France, Table S3: Other dishes/foods used for Principal Component Analysis, Table S4: Discrimination measures on main dimensions (eigen values > 1) of Multiple Factorial Correspondence Analyses 1, Table S5: Discrimination measures on main dimensions (eigen values > 1) of Multiple Factorial Correspondence Analyses 2, Table S6: Discrimination measures on main dimensions (eigen values > 1) of Multiple Factorial Correspondence Analyses 3, Table S7: Discrimination measures on main dimensions (eigen values > 1) of Multiple Factorial Correspondence Analyses 4, Table S8: Factor loadings of principal components above the elbow on the screen plot,
