# Supplementary material for: Nutrition Transition and Biocultural Determinants of Obesity among Cameroonian Migrants in Urban Cameroon and France
Source: Int J Environ Res Public Health. 2017 Jun 29;14(7):696. doi: 10.3390/ijerph14070696 (PMC5551134; doi:10.3390/ijerph14070696)
Supplement: Supplementary file 1 [file ijerph-14-00696-s001.pdf]

Supplementary Materials: Nutrition Transition and Biocultural Determinants of Obesity among Cameroonian Migrants in Urban Cameroon and France

Emmanuel Cohen, Norbert Amougou, Amandine Ponty, Juliette Loinger-Beck, Téodyl Nkuintchua, Nicolas Monteillet, Jonathan Y. Bernard, Rihlat Saïd-Mohamed, Michelle Holdsworth and Patrick Pasquet

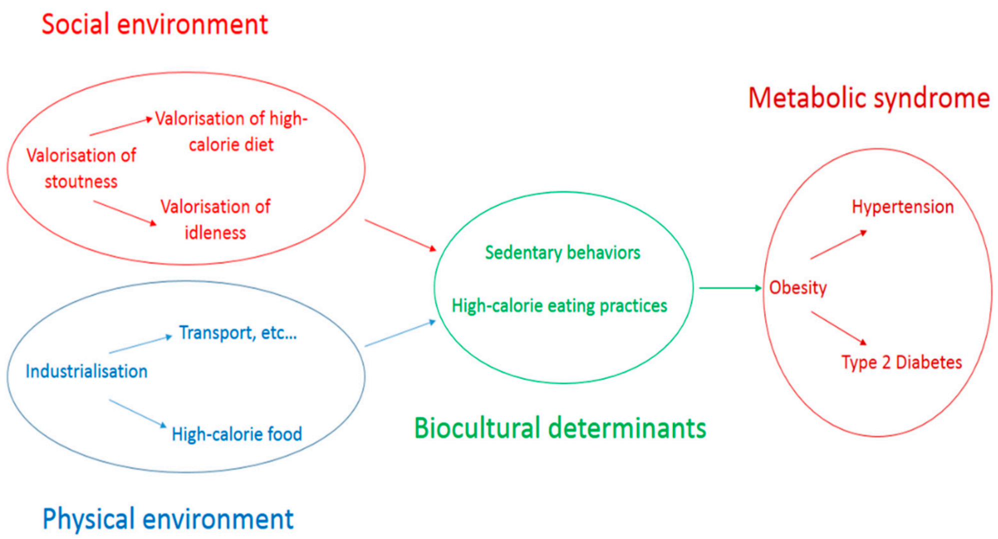

Figure S1. Biocultural determinants of obesity among Cameroonian migrants.

Table S1. List and consumption frequency of main dishes/foods in Cameroon.

**Cameroon**	**Energy Value** [1]				**Food Frequency** [2]		
100 g dry matter							
Modern food	Kcal	Lipid	Glucid	Protein	Rural Cameroon	Urban Cameroon	*p*
Roasted chicken	263.8	19.8	0	21.4	M: 73.0; W: 61.4	M: 85.7; W: 79.3	***
Braised chicken	263.8	19.8	0	21.4	M: 53.2; W: 47.0	M: 77.9; W: 75.4	***
Braised pork	519.5	49.9	0	17.6	M: 52.4; W: 39.4	M: 77.9; W: 71.5	***
Braised fish	249.8	15	0	28.7	M: 74.6; W: 67.4	M: 92.1; W: 90.0	***
Meat skewers	281.1	19.9	0	25.5	M: 50.0; W: 28.8	M: 75.0; W: 68.7	***
Red beans							
(“Jazz sauce”)	188.2	7.3	21.8	8.9	M: 80.1; W: 82.6	M: 88.0; W: 91.6	**
Fried rice	160	3.2	30.1	2.6	M: 93.6; W: 93.1	M: 96.4; W: 96.1	NS
Mayonnaise	761.6	83.6	0.3	2	M: 44.0; W: 33.1	M: 68.6; W: 69.3	***
Cheese	282.7	22.7	2.8	16.8	M: 18.4; W: 14.6	M: 38.6; W: 40.2	***
Margarine	746.9	82.5	0.3	0.8	M: 50.4; W: 47.7	M: 57.9; W: 71.5	***
Sugar [3]	400.0	0	100.0	0	M: 89.5; W: 91.6	M: 93.6; W: 89.4	NS
Chocolate	544	32	56.5	7.5	M: 58.4; W: 56.9	M: 66.4; W: 77.1	***
Sandwich	293.2	13.6	31.9	10.8	M: 92.1; W: 94.7	M: 95.7; W: 97.8	*
Juices	38.1	0.1	8.7	0.6	M: 59.2; W: 78.5	M: 72.9; W: 78.8	*
Beer	56.2	0	3.5	0.4	M: 78.4; W: 66.2	M: 87.9; W: 82.7	***
Whisky	241.0	0	0	0	M: 50.4; W: 16.9	M: 60.7; W: 40.2	***
Traditional food	Kcal	Lipid	Glucid	Protein	Rural Cameroon	Urban Cameroon	
“Koki leaves/							
peanuts pistachios”	114.1	10.1	5.7	3.8	M: 33.3; W: 28.0	M: 12.9; W: 14.5	***
Crushed bananas/							
beans	252.2	7.3	36.6	10.1	M: 74.6; W: 84.1	M: 53.6; W: 53.1	***

Bean pie	165.9	7.2	17.5	7.9	M: 94.4; W: 94.7	M: 73.6; W: 76.0	***
"Yellow gombo							
sauce" [4]	120.0	10.3	5.0	1.9	M: 80.2; W: 88.6	M: 83.6; W: 78.2	NS
Crushed plantains/							
beans	252.2	7.3	36.6	10.1	M: 78.6; W: 78.0	M: 66.4; W: 71.5	*
Crushed apples/							
Beans [4]	252.2	7.3	36.6	10.1	M: 82.5; W: 72.7	M: 74.3; W: 76.5	NS
Corn peanuts/							
Beans	182.1	7.8	22.2	5.8	M: 47.6; W: 56.1	M: 45.0; W: 40.8	*

[1] Foods/dishes energy contents from FPPB (Amougou et al., in prep) and SUVIMAX (2006) [89]; [2] At least one to three times per month; [3] Urban subjects have a higher consumption than rural subjects in the total sample; [4] Rural subjects have a higher consumption than urban subjects in the total sample; M: Men; W: Women; Chi2 between the two samples, men and women aggregated: * $p < 0.05$; ** $p < 0.01$; *** $p < 0.001$.

Table S2. List and consumption frequency of main high calorie dishes/foods in France.

**France**	**Energy Value [1]**				**Food Frequency [2]**		
100 g dry matter							
Modern food	Kcal	Lipid	Glucid	Protein	Migrants settled	New migrants	*p*
Sandwich	337.5	16.3	31.5	16.2	M: 58.3; W: 28.6	M: 75.0; W: 62.5	*
Hamburger	270.1	12.1	27.6	12.7	M: 58.3; W: 14.3	M: 75.0; W: 50.0	*
Pizza	221.1	10.7	21.7	9.5	M: 66.7; W: 28.6	M: 81.3; W: 62.5	*
Kebab	203.7	6.5	26.2	10.1	M: 25.0; W: 14.3	M: 50.0; W: 50.0	*
Jam	274.0	0	68	0.5	M: 33.3; W: 28.6	M: 56.3; W: 50.0	NS
Butter [3]	747.3	82.5	0.5	0.7	M: 83.3; W: 71.4	M: 100.0; W: 50.0	NS
Chocolate	544.0	32	56.5	7.5	M: 74.6; W: 57.1	M: 92.1; W: 100.0	*
Cake [3]	289.5	14.7	34.5	4.8	M: 66.7; W: 85.7	M: 87.5; W: 75.0	NS
Sugar [3]	400.0	0	100	0	M: 100.0; W: 64.3	M: 93.8; W: 75.0	NS
Juices	38.1	0.1	8.7	0.6	M: 75.0; W: 64.3	M: 100.0; W: 100.0	**
Soda	40.0	0	10	0	M: 50.0; W: 35.7	M: 81.3; W: 75.0	**
Beer	56.2	0	3.5	0.4	M: 50.0; W: 21.4	M: 68.8; W: 75.0	*
Alcohol (Vodka)	238.0	0	0	0	M: 50.0; W: 42.9	M: 68.8; W: 62.5	NS
Traditional food	Kcal	Lipid	Glucid	Protein	French youth	Cameroonian youth	
"Ndolé"	176.8	13.8	6.9	6.3	M: 85.7; W: 90.0	M: 78.6; W: 75.0	NS
"Kondré"	136.5	6.9	17.4	1.2	M: 92.9; W: 90.0	M: 57.1; W: 33.3	***
Gombo [4]	90.0	6.9	5.0	1.9	M: 50.0; W: 80.0	M: 64.3; W: 58.3	NS
Peanut sauce [4]	101.8	9.8	1.5	1.8	M: 85.7; W: 60.0	M: 71.4; W: 66.7	NS
Pistachio sauce [4]	99.1	9.9	0.8	1.8	M: 71.4; W: 70.0	M: 78.6; W: 50.0	NS
"Koki"	251.3	7.2	36.6	10.1	M: 57.1; W: 80.0	M: 42.9; W: 41.7	NS
"Director general							
chicken"	343.3	26.7	3.5	22.2	M: 71.4; W: 60.0	M: 42.9; W: 25.0	*
"Yellow sauce"	120.0	10.3	5.0	1.9	M: 42.9; W: 70.0	M: 35.7; W: 41.7	NS

[1] Foods/dishes energy contents from FPPB (Amougou et al., in prep) and SUVIMAX (2006) [89]; [2] At least one to three times per month; [3] New Migrants have a higher consumption than settled migrants in the total sample; [4] Migrants with a French youth have a higher consumption than migrants with a Cameroonian youth in the sample; M: Men; W: Women; Chi2 between the two samples, men and women aggregated: * $p < 0.05$; ** $p < 0.01$; *** $p < 0.001$.

Table S3. Other dishes/foods used for Principal Component Analysis.

**Food variables**	**Energy Value [1]**			
100 g dry matter	Kcal	Lipid	Glucid	Protein
Peanut gombo sauce	129.4	10.1	6.1	3.6
Crushed vegetables	252.2	7.3	36.6	10.1
Peanuts beans	165.9	7.2	17.5	7.9
Palm beans	188.2	7.3	21.8	8.9
Mbongo	135.7	10.0	7.5	4.0
Nkwi	216.7	2.3	14.5	34.5

Dairy products	33.4	0.2	4.6	3.3
Milk	62.7	3.5	4.6	3.2
Plantain	128.7	0.3	30.5	1.1
Bread	275.0	1.3	57.4	8.5

Table S4. Discrimination measures on main dimensions (eigen values > 1) of Multiple Factorial Correspondence Analyses 1.

**Variables**	**Dimensions**				
	**1**	**2**	**3**	**4**	**5**
Living area	**0.331**	0.072	0.155	0.003	0.053
Urban duration	0.087	0.127	0.340	0.002	0.138
Red beans	0.087	0.009	0.025	0.197	0.250
Sandwich	0.051	0.131	0.010	0.042	0.171
Braised fish	**0.278**	0.008	0.025	0.054	0.073
Braised pork	**0.400**	0.058	0.049	0.027	0.002
Braised chicken	**0.339**	0.081	0.059	0.033	0.000
Roasted chicken	0.101	0.077	0.061	0.008	0.064
Meat skewers	**0.414**	0.068	0.001	0.005	0.057
Fried rice	0.050	0.066	0.056	0.007	0.002
Sugar	0.072	0.185	0.050	0.043	0.076
Beer	0.073	0.139	0.074	0.256	0.005
Juices	0.098	0.252	0.049	0.000	0.041
Whisky	0.119	0.075	0.047	0.364	0.009
Margarine	**0.311**	0.184	0.040	0.011	0.020
Mayonnaise	**0.330**	0.042	0.097	0.040	0.052
Cheese	**0.263**	0.052	0.085	0.006	0.036
Chocolate	**0.258**	0.187	0.001	0.005	0.008
Active Total	3.662	1.813	1.224	1.105	1.056

In bold, discrimination measures > 0.2 on dimension 1.

Table S5. Discrimination measures on main dimensions (eigen values > 1) of Multiple Factorial Correspondence Analyses 2.

**Variables**	**Dimensions**		
	**1**	**2**	**3**
Living area	**0.296**	0.333	0.029
Urban duration	**0.218**	0.341	0.012
Yellow gombo sauce	0.139	0.122	0.001
Crushed plantains_beans	**0.310**	0.173	0.202
Bean pie	**0.335**	0.004	0.309
Crushed bananas_beans	**0.441**	0.048	0.200
Corn_peanuts beans	0.178	0.059	0.003
Crushed apples_beans	**0.274**	0.129	0.295
Koki leaves_Peanuts pistachios	0.163	0.038	0.017
Want to gain weight	0.088	0.098	0.003
Active Total	2.443	1.345	1.070

In bold, discrimination measures > 0.2 on dimension 1.

Table S6. Discrimination measures on main dimensions (eigen values > 1) of Multiple Factorial Correspondence Analyses 3.

**Variables**	**Dimensions**				
	**1**	**2**	**3**	**4**	**5**
Migrant status	**0.360**	0.027	0.138	0.027	0.165
Youth living area	0.064	0.161	0.260	0.061	0.144
Hamburger	**0.528**	0.100	0.135	0.008	0.009
Sandwich	**0.575**	0.051	0.045	0.011	0.000

Kebab	**0.313**	0.147	0.007	0.026	0.255
Pizza	**0.633**	0.053	0.141	0.034	0.003
Butter	**0.292**	0.043	0.017	0.310	0.122
Cake	0.174	0.141	0.014	0.003	0.226
Chocolate	**0.238**	0.324	0.124	0.018	0.016
Jam	0.176	0.219	0.005	0.143	0.017
Sugar	0.053	0.098	0.006	0.442	0.000
Juices	0.219	0.237	0.024	0.247	0.000
Soda	0.415	0.015	0.073	0.062	0.132
Beer	0.127	0.130	0.437	0.012	0.006
Alcohol	0.117	0.241	0.281	0.035	0.073
Active Total	4.284	1.988	1.708	1.437	1.168

In bold, discrimination measures > 0.2 on dimension 1.

Table S7. Discrimination measures on main dimensions (eigen values>1) of Multiple Factorial Correspondence Analyses 4.

**variables**	**Dimensions**			
	**1**	**2**	**3**	**4**
Migrant status	0.100	0.076	0.445	0.113
Youth living area	**0.247**	0.001	0.452	0.084
Stoutness perception	0.087	0.052	0.044	0.358
Ndole	**0.488**	0.208	0.029	0.038
Gombo	**0.263**	0.123	0.035	0.284
Koki	**0.440**	0.082	0.000	0.022
Kondre	**0.601**	0.000	0.031	0.025
Director General chicken	**0.474**	0.008	0.015	0.095
Yellow sauce	**0.345**	0.315	0.019	0.002
Pistachio sauce	0.177	0.183	0.136	0.075
Peanut sauce	0.116	0.436	0.066	0.090
Active Total	3.338	1.484	1.271	1.186

In bold, discrimination measures > 0.2 on dimension 1.

Table S8. Factor loadings of principal components above the elbow on the screen plot.

**Variables**	Factor 1	Factor 2	Factor 3
Ndolé	−0.163	0.286	−0.025
Mbongo	**−0.484**	0.245	0.034
Nkwi	−0.0829	0.559	−0.120
Koki	**−0.352**	0.237	−0.169
Kondre	**−0.308**	0.309	0.128
Director General chicken	**−0.606**	0.032	0.267
Yellow Gombo Sauce	−0.150	0.518	0.020
Pistachio Sauce	−0.290	0.292	0.191
Peanut Gombo Sauce	−0.283	0.368	−0.067
Crushed vegetables	−0.032	0.242	−0.411
Peanuts Beans	**−0.414**	0.194	−0.018
Palm Beans	−0.048	0.483	−0.277
Bread	−0.128	0.039	−0.256
Fried Rice	−0.224	−0.180	−0.177
Red beans	**−0.350**	0.253	0.092
Sandwich	−0.187	0.317	−0.449
Braised fish	**−0.494**	−0.134	0.059
Braised pork	**−0.617**	−0.030	0.297
Braised chicken	**−0.550**	−0.138	0.367
Meat skewers	**−0.610**	0.102	0.251
Sugar	−0.293	−0.068	−0.433
Beer	−0.239	0.226	0.196
Juices	−0.294	−0.196	−0.437
Whisky	−0.297	−0.005	0.255

Margarine	**−0.535**	−0.254	−0.351
Dairy products	**−0.468**	−0.481	−0.032
Milk	**−0.506**	−0.220	−0.104
Cheese	**−0.438**	−0.511	−0.053
Chocolate	**−0.460**	−0.222	−0.375
Plantain	−0.249	0.088	−0.035

In bold, factor loadings > 0.3 on principal component 1.

© 2017 by the authors; licensee MDPI, Basel, Switzerland. This article is an open access article distributed under the terms and conditions of the Creative Commons by Attribution (CC-BY) license (http://creativecommons.org/licenses/by/4.0/).
